# Imatinib in systemic mastocytosis: a phase IV clinical trial in patients lacking exon 17 *KIT* mutations and review of the literature

**DOI:** 10.18632/oncotarget.10711

**Published:** 2016-07-19

**Authors:** Iván Alvarez-Twose, Almudena Matito, José Mário Morgado, Laura Sánchez-Muñoz, María Jara-Acevedo, Andrés García-Montero, Andrea Mayado, Carolina Caldas, Cristina Teodósio, Javier Ignacio Muñoz-González, Manuela Mollejo, Luis Escribano, Alberto Orfao

**Affiliations:** ^1^ Instituto de Estudios de Mastocitosis de Castilla La Mancha (CLMast), Hospital Virgen del Valle, Toledo, Spain; ^2^ Centro de Investigación del Cáncer/IBMCC (USAL/CSIC) and IBSAL, Departamento de Medicina and Servicio General de Citometría, University of Salamanca, Salamanca, Spain; ^3^ Department of Immunology, Erasmus Medical Center, University of Rotterdam, Rotterdam, The Netherlands; ^4^ Department of Pathology, Hospital Virgen de la Salud, Toledo, Spain; ^5^ Spanish Network on Mastocytosis (REMA), Toledo and Salamanca, Spain

**Keywords:** mast cell, mastocytosis, well-differentiated systemic mastocytosis, imatinib, KIT

## Abstract

Resistance to imatinib has been recurrently reported in systemic mastocytosis (SM) carrying exon 17 *KIT* mutations. We evaluated the efficacy and safety of imatinib therapy in 10 adult SM patients lacking exon 17 *KIT* mutations, 9 of which fulfilled criteria for well-differentiated SM (WDSM). The World Health Organization 2008 disease categories among WDSM patients were mast cell (MC) leukemia (*n* = 3), indolent SM (*n* = 3) and cutaneous mastocytosis (*n* = 3); the remainder case had SM associated with a clonal haematological non-MC disease. Patients were given imatinib for 12 months −400 or 300 mg daily depending on the presence vs. absence of > 30% bone marrow (BM) MCs and/or signs of advanced disease–. Absence of exon 17 *KIT* mutations was confirmed in highly-purified BM MCs by peptide nucleic acid-mediated PCR, while mutations involving other exons were investigated by direct sequencing of purified BM MC DNA. Complete response (CR) was defined as resolution of BM MC infiltration, skin lesions, organomegalies and MC-mediator release-associated symptoms, plus normalization of serum tryptase. Criteria for partial response (PR) included ≥ 50% reduction in BM MC infiltration and improvement of skin lesions and/or organomegalies. Treatment was well-tolerated with an overall response rate of 50%, including early and sustained CR in four patients, three of whom had extracellular mutations of *KIT*, and PR in one case. This later patient and all non-responders (*n* = 5) showed wild-type *KIT*. These results together with previous data from the literature support the relevance of the *KIT* mutational status in selecting SM patients who are candidates for imatinib therapy.

## INTRODUCTION

Mastocytosis is a rare and heterogeneous disease characterized by an expansion of clonal mast cells (MCs) in different organs and tissues such as the bone marrow (BM), skin, gastrointestinal tract, liver, spleen and lymph nodes. Based on the World Health Organization (WHO) 2008 classification, mastocytosis is subclassified into several categories including cutaneous mastocytosis (CM), and systemic mastocytosis (SM). SM is further subdivided into indolent SM (ISM), aggressive SM (ASM), SM associated with a clonal haematological non-MC disease (SM-AHNMD), MC leukemia (MCL), MC sarcoma and a provisional subvariant of ISM termed smoldering SM (SSM) [1]. Most SM patients (∼94%) show morphologically abnormal CD25^+^ BM MCs [2, 3] that carry *KIT* mutations [4–7], from which the *KIT* D816V is the most common one (∼90% of SM cases) [6]. The later mutation involves the tyrosine kinase (TK) 2 domain of KIT and leads to constitutive ligand-independent activation of the KIT receptor [8]. Other mutations involving either exon 17 or other exons of *KIT* (e.g. exons 8, 9, 10 and 11), or a wild-type KIT receptor can be detected in a small percentage of SM patients (∼3% and ∼6%, respectively) [6, 9–18]. Of note, many of these D816V-negative patients correspond to well-differentiated SM (WDSM), a recently described rare subvariant of SM defined by skin involvement associated with clonal expansion of mature-appearing, CD25^−/low^ MCs in the BM, for which specific diagnostic criteria have been proposed [19] and adopted in the new WHO 2016 classification. Of note, a significant proportion of patients with indolent forms of WDSM fail to fulfill the current WHO 2008 diagnostic criteria for SM, despite they systematically show features of a systemic MC disease such as BM MC aggregates, aberrant CD30 expression on BM MCs, mutations involving any region of *KIT* and/or a clonal nature based on the HUMARA pattern of inactivation of the X chromosome.

In the last decade, TK inhibitor (TKI) targeted-therapy (e.g. imatinib mesylate) has become the front-line treatment for several TK-driven diseases such as chronic myeloid leukemia (CML) [20], gastrointestinal stromal tumor (GIST) [21] and chronic eosinophilic leukemia (CEL)/hypereosinophilic syndrome (HES) [22]. The outstanding clinical responses obtained in these diseases have led to explore the potential utility of TKI also in mastocytosis. Data from single case reports and small series of mastocytosis patients treated with imatinib prompted the U.S. Food and Drug Administration (FDA) to approve in 2006 its use in adults with ASM lacking the D816V *KIT* mutation or with unknown *KIT* mutational status. Since then, the number of reports about D816V-negative patients showing no response to imatinib has significantly increased, while the responding cases among D816V-negative patients include both cases with ASM as well as patients with non-advanced forms of mastocytosis.

Here we report the results of an investigator-initiated clinical trial that evaluated the efficacy of imatinib targeted therapy in 10 patients with SM −9 patients with WDSM (including 3 CM, 3 ISM and 3 MCL) and one SM-AHNMD– lacking mutations at exon 17 of the *KIT* gene selected from 453 consecutive patients diagnosed with SM. Due to the rarity of these SM cases lacking exon 17 *KIT* mutations (≤ 4%) [6], a critical review of mastocytosis cases treated with imatinib who have been reported in the literature is also provided to better estimate the response rates to imatinib according to the *KIT* mutational status.

## RESULTS

### Clinical presentation

**Figure 1:**
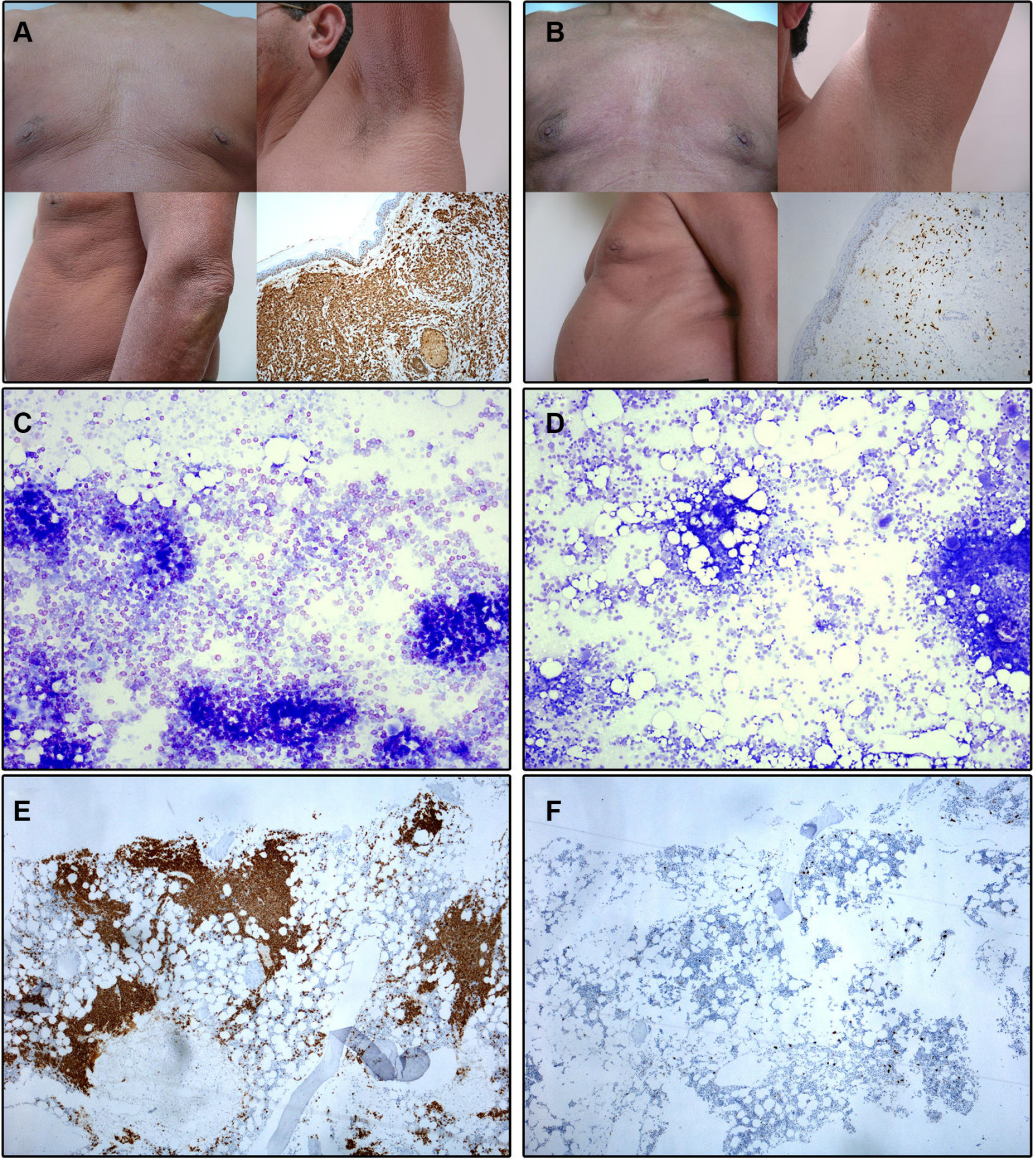
Illustrating skin and bone marrow microscopic images obtained before and at month +12 of imatinib therapy in a patient with advanced WDSM who achieved CR (case #1) (**A**–**B**) Macroscopic appearance of the skin and histological findings of skin biopsy (tryptase stain, 100x magnification) at diagnosis (A) and after imatinib therapy (B). (**C**–**D**) BM smears (toluidine blue stain, 100x magnification) at diagnosis (C) and after imatinib therapy (D). (**E**–**F**) BM sections (c-kit stain, 100x magnification) at diagnosis (E) and after imatinib therapy (F).

Overall, 9/10 patients had WDSM according to recently proposed criteria [19] consisting of histologically-proven mastocytosis in the skin and BM compact aggregates of mature-appearing CD25^−/low^/CD2^−/low^ MCs, together with clusters of ≥ 2 MCs outside BM particles (*n* = 9), aberrant expression of CD30 and/or overexpression of cytoplasmic proteases (*n* = 4), mutations involving exons other than exon 17 of *KIT* (*n* = 3), a clonal HUMARA pattern of inactivation of X chromosome (*n* = 3), and/or female sex with either pediatric disease onset (*n* = 7) and/or familial aggregation (*n* = 4). According to the WHO 2008 classification [1], these 9 patients were subclassified as having MCL (*n* = 3), ISM (*n* = 3) and CM (*n* = 3) (Table 1). Except for a patient who presented with an adult-onset mastocytoma in her right arm, all other WDSM patients referred pediatric-onset of mastocytosis-associated maculopapular skin lesions in 5 cases and diffuse cutaneous mastocytosis lesions with generalized thickening of the skin in the other 3 patients (Table 1 and Figure 1). Of note, these three later patients included two sisters and their father, who had also an associated GIST.

**Table 1:** Clinical, biological and molecular characteristics at diagnosis and response to imatinib therapy of the 10 patients included in this study

Findings	Case #1	Case #2	Case #3	Case #4	Case #5	Case #6	Case #7	Case #8	Case #9	Case #10
**Sex**	M	F	F	M	F	F	F	F	F	F
**Age at onset/diagnosis‡**	Birth/55 y	3 mo/27 y	3 mo/20 y	32 y/37 y	1 y/24 y	4 y/26 y	14 y/21 y	2 y/26 y	10 mo/21 y	60 y/69 y
**Skin lesions**	DCM	DCM	DCM	No	MPCM*	MPCM*	MPCM*	MPCM*	MPCM*	Cutaneous mastocytoma
**Baseline MC-mediator release symptoms**	P,GI,D	P,Fl,GI,D	P,D	No	Fl,A	P	P,F	P,Fl,GI,D,A	P,Fl,GI	Fl,A
**BM MC aggregates**	Yes	Yes	Yes	Yes	Yes	Yes	Yes	Yes	Yes	Yes
**>20% of MCs in BM smears**	Yes	Yes	No	No	No	No	No	No	No	Yes
**BM MC morpholgy**	Normal	Normal	Normal	Abnormal	Normal	Normal	Normal	Normal	Normal	Normal
**BM MC phenotype**	CD2^−^ CD25^−^ CD30^+^	CD2^−^ CD25^−^ CD30^+^	CD2^−^ CD25^−^ CD30^+^	CD2^−^ CD25^+bright^	CD2^−^ CD25^−^	CD2^−^ CD25^−^	CD2^−^ CD25^−^	CD2^+low^ CD25^+low^ CD30^−^	CD2^+low^ CD25^−^ CD30^−^	CD2^+low^ CD25^−^ CD30^−^
**KIT mutation**	K509I	K509I	K509I	Negative	Negative	Negative	Negative	Negative	Negative	Negative
**HUMARA**	NA	NA	Polyclonal	NA	Clonal	Polyclonal	Clonal	Clonal	Polyclonal	Polyclonal
**Organomegalies**	No	No	No	S	L	No	No	No	L	No
**C-findings**	No	No	No	No	No	No	No	No	No	No
**Associated diseases**	GIST	GIST	GIST	CEL	No	No	No	No	No	No
**WHO diagnostic subtype**	MCL	MCL	ISM	SM-AHNMD	CM	CM	CM	ISM	ISM	MCL
**WDSM criteria** [19]	Yes	Yes	Yes	No	Yes	Yes	Yes	Yes	Yes	Yes
**Advanced MC disease†**	Yes	Yes	No	Yes	No	No	No	No	No	Yes
**Prior therapies**	DCG,H1	DCG,H1	DCG,H1	HU,CS	DCG,H1	H1	H1	DCG,H1	DCG,H1	DCG,H1, H2,LTA
**Imatinib dosage (mg/day)**										
Initial prescribed dosage	400	400	400	400	300	300	300	300	400	400
Dosage reduction	No	300	300	300	No	No	No	No	No	300
**BM MCs by IHC (%)**										
Before imatinib	60	80	20	15	5	5	5	5	40	65
+6 mo	< 1	< 1	< 1	< 1	NA	NA	NA	NA	40	80
+12 mo	< 1	< 1	< 1	< 1	3	5	5	5	NA	NA
**BM MCs by FC (%)**										
Before imatinib	7	13	0.78	0.28	0.01	0.02	0.01	0.06	0.4	14
+6 mo	0.02	0.007	0.0009	0.0009	NA	NA	NA	NA	0.4	19
+12 mo	0.01	0.003	0.0005	0.0007	0.005	0.03	0.03	0.09	NA	NA
**sT levels (μg/L)**										
Before imatinib	90.9	126	43.8	46.9	18.1	11.0	8.6	16.7	386	196
+6 mo	3.2	4.9	1.6	2.3	14.7	9.6	4.8	10.5	385	244
+12 mo	1.6	3.1	1.4	1.8	14.7	9.5	4.8	8.9	NA	NA
**Response to therapy** **(+6 mo/+12 mo)**	CR/CR	CR/CR	CR/CR	CR/CR	NA/PR	NA/NR	NA/NR	NA/NR	NR/NA	NR/NA

M, male; F, female; y, years; mo, months; DCM, diffuse cutaneous mastocytosis; MPCM, maculopapular cutaneous mastocytosis; P, pruritus; Fl, flushing; GI, gastrointestinal pain; D, diarrhea; A, anaphylaxis; BM, bone marrow; MC, mast cell; HUMARA, human androgen receptor assay; NA, not assessed/applicable; S, splenomegaly; L, hepatomegaly; GIST, gastrointestinal stromal tumor; CEL, chronic eosinophilic leukemia; WHO, World Health Organization; MCL, mast cell leukemia; ISM, indolent systemic mastocytosis;; SM-AHNMD, systemic mastocytosis with associated clonal hematological non-MC disease; CM, cutaneous mastocytosis; WDSM, well-differentiated systemic mastocytosis; DCG, disodium cromoglycate; H1, H1 blockers; HU, hydroxyurea; CS, corticosteroids; H2, H2 blockers; LTA, leukotriene antagonists; IHC, immunohistochemistry; FC, flow cytometry; sT, serum tryptase; CR, complete response; PR, partial response; NR, no response.

‡Age at onset was defined as that at which mastocytosis-associated features (e.g. increased serum tryptase for patient #4 and emergence of skin lesions for the remaining patients) were first noticed, while age at diagnosis as that at which systemic disease was confirmed by BM studies.

*These cases corresponded to the monomorphic variant of MPCM according to a novel consensus classification of CM proposed by the ECNM [24].

†Defined as the presence of C-findings, > 20% of MCs in BM smears and/or an associated clonal non-MC lineage haematological disease.

**Figure 2:**
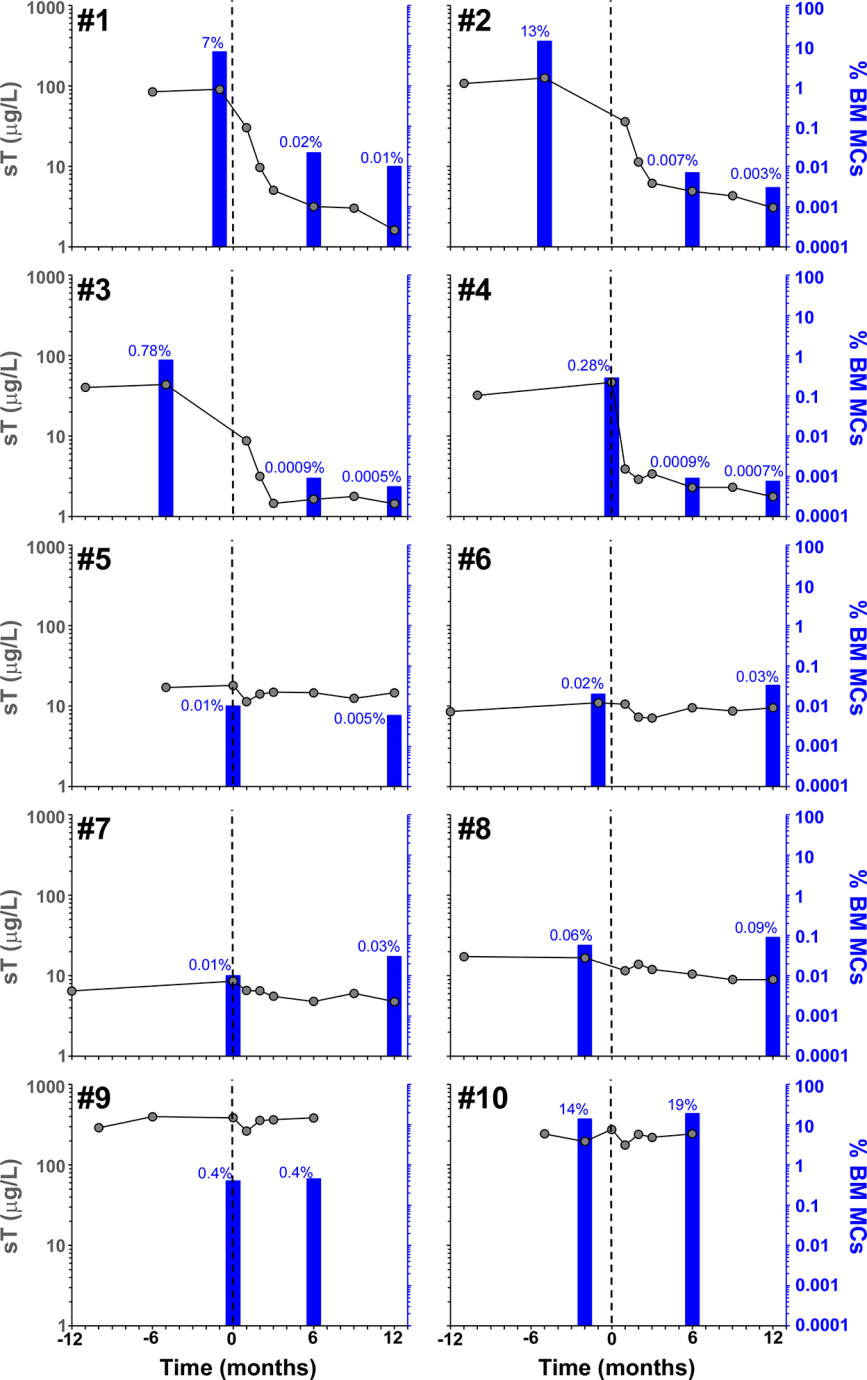
Effect of imatinib therapy on BM MC counts and sT levels in the 10 patients included in the present clinical trial Vertical dotted lines indicate start of imatinib therapy, blue bars and grey dots represent the percentage of pathologic/aberrant BM MCs as assessed by flow cytometry and sT values, respectively, before and after imatinib therapy.

The remainder patient was a 37 year-old male with neither mastocytosis skin lesions nor MC-mediator release symptoms who had a 5-year history of sustained eosinophilia and increased serum tryptase (sT) levels. At referral, he showed a peripheral blood (PB) eosinophil count of 5.2 × 10^9^/L, increased sT levels (32 μg/L) and splenomegaly (17.5 cm). BM aspirate and biopsy analyses revealed a hypercellular marrow with increased eosinophils together with the presence of CD25^+bright^, spindle-shaped MCs forming compact aggregates consistent with the diagnosis of ISM-AHNMD, the associated haematological disorder being a CEL (ISM-CEL).

**Figure 3:**
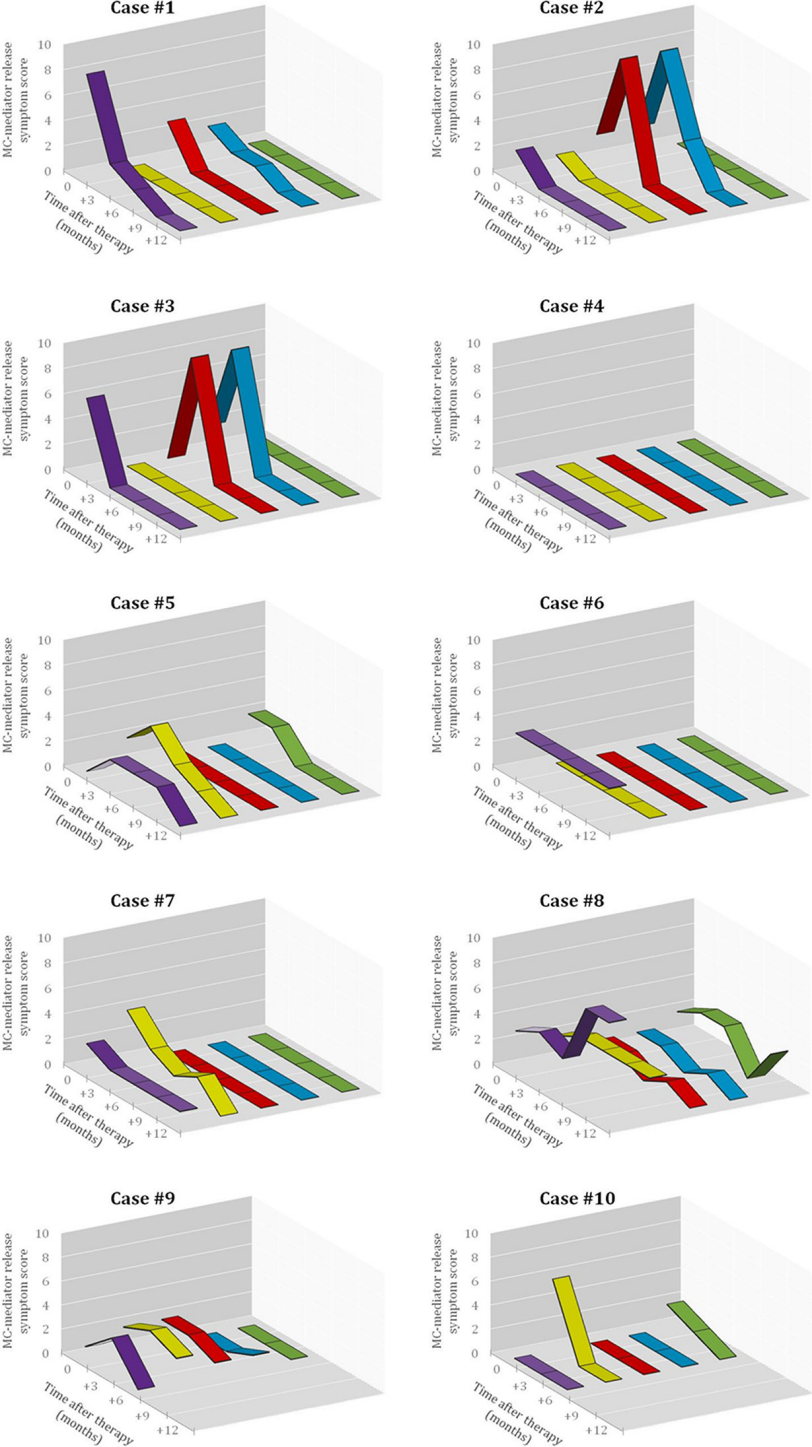
Effect of imatinib therapy on MC-mediator release associated symptoms in the 10 patients included in the present clinical trial Symptomatic response was evaluated in each patient before starting imatinib and every 3 months thereafter using Likert-type scales obtained from specific questionnaires designed by the REMA, by which MC-mediator release symptoms (e.g. pruritus, purple lane; flushing, yellow lane; abdominal cramping, red lane; diarrhea, blue lane; and anaphylaxis, green lane) were graded as described in detail in the Methods section. The X-axis represents the different time-points at which the questionnaires were collected, while the Y-axis represents the overall score (i.e. the frequency score multiplied by the severity score) for each MC-mediator release-associated symptom.

At diagnosis, the most frequent MC-mediator release-associated symptoms were: pruritus (*n* = 7), flushing (*n* = 6), abdominal cramping (*n* = 4), diarrhea (*n* = 4) and anaphylaxis (*n* = 3) (Table 1 and Figure 3). Only 2/10 patients had bone mass loss consisting of osteopenia (case #5) and osteoporosis (case #10).

Overall, 6 patients (3 advanced WDSM, 2 indolent WDSM and the ISM-CEL case) received an initial dose of imatinib of 400 mg imatinib per day, while the other 4 WDSM patients received 300 mg imatinib per day (Table 1).

### KIT mutational status and additional genetic studies

As per the inclusion criteria, none of the 10 patients showed mutations involving exon 17 of *KIT*. Further sequencing of other *KIT* exons revealed a germline K509I mutation (exon 9) in the 3 familial WDSM patients (Table 1). In the SM-CEL patient, Janus kinase 2 (*JAK-2*) gene mutations, rearrangements of the platelet-derived growth factor receptor (*PDGFR*) α and β genes, and T-cell clonality were ruled out, while genetic analyses for the fibroblast growth factor receptor 1 (*FGFR1*) gene were not performed.

### Toxicity

Imatinib was reduced from 400 mg to 300 mg in 2 patients (cases #2 and #3) due to grade 3 gastrointestinal symptoms. In another two patients receiving 400 mg/day (cases #4 and #10), treatment was transiently stopped due to grade 4 neutropenia and grade 4 anemia which were successfully recovered with G-CSF support and red blood cell transfusions, respectively; afterward, imatinib was reintroduced in both cases at 300 mg/day with no further haematological toxicity. Grade ≤ 2 adverse events included: muscle cramps (70%), nausea (50%), edema (40%), skin rash (20%), alopecia (20%), dyspepsia (10%) and abdominal pain (10%).

### Response to therapy

Overall, objective responses were obtained in 5/10 patients including CR in 4 of the 5 responder patients (2 advanced WDSM, 1 indolent WDSM and the SM-CEL patient), and PR in another indolent WDSM case (Table 1). All 4 patients who achieved CR showed complete clearance of BM MC infiltrates (together with negative expression of CD25 on BM MCs and a dramatic decrease of BM eosinophil counts in the SM-CEL patient) at month +6 of therapy; in all four cases, CR was maintained at month +12 of therapy (Table 1 and Figures 1 and 2). In parallel, all four CR cases also showed early normalization of sT levels (Table 1 and Figure 2), as well as complete disappearance of MC-mediator release-associated symptoms (Figure 3) and gradual improvement of skin lesions in those 3 patients who had cutaneous involvement, as histologically confirmed at the end of the study (Figure 1). Noteworthy, treatment with imatinib was continued beyond the end of the study in these 3 later patients because of the presence of an associated GIST. At last follow-up (e.g. 72 months after initiation of imatinib in case #1 and 66 months after starting on imatinib in cases #2 and #3), all three patients still showed sT levels < 5 μg/L, normal skin appearance and neither MC-mediator release symptoms nor drug-associated long-term side effects; although no further BM studies were performed, these findings suggest that they remained in continuous CR of their SM. In turn, repeated CT scans during the follow-up period showed no significant changes of GIST lesions in these three patients. In the SM-CEL case, the PB eosinophil count decreased to 0.1 × 10^9^/L after 4 weeks of therapy and remained below 0.4 × 10^9^/L thereafter; in addition, splenomegaly had disappeared at the end of the study. In view of these results imatinib was discontinued, and both eosinophil counts as well as sT levels remained within normal range for 5 additional years. Of note, in 3/4 CR patients, *KIT* mutations in exon 9 had been detected (Table 1).

The only patient who attained PR showed 50% decrease of BM MCs by flow cytometry (Table 1 and Figure 2) in the absence of BM MC aggregates, together with resolution of pre-existing (mild) hepatomegaly. In parallel, sT levels decreased by 20% (Table 1 and Figure 2), MC-mediator release-associated symptoms completely disappeared (Figure 3) and modest fading of skin lesions was observed at month +12. The patient kept under imatinib treatment for one additional year, during which no further MC-mediator release-associated symptoms occurred and both sT levels and skin lesions remained stable; thereafter, she decided to get pregnant and thereby, treatment was discontinued.

Among the 5 non-responders, imatinib was discontinued at month +6 of therapy in two patients with advanced SM due to lack of response upon BM re-evaluation (Table 1 and Figure 2). Despite this, both patients showed improvement of symptoms at that time (Figure 3). In the remaining 3 patients who completed the trial, neither a significant reduction of BM MC numbers (Table 1 and Figure 2), nor changes in skin lesions were observed. Despite this, sT levels decreased in all 3 patients by 13%, 44% and 46% (Table 1 and Figure 2), in parallel to a mild decrease of MC-mediator release-associated symptoms in one patient (Figure 3). All five refractory patients had wild-type *KIT* (Table 1).

## DISCUSSION

Currently, SM remains an incurable disease. SM patients are typically managed with drugs aimed at improving and/or preventing symptoms related to the release of MC mediators, together with cytoreductive therapy in advanced cases (e.g. ASM, MCL and SM-AHNMD). Interferon-α and cladribine are the most commonly used cytotoxic agents to decrease MC tumor load in advanced SM patients; unfortunately, objective responses occur in only 20–30% of cases and these typically consist of transient PR [25, 26].

In the last decade, TKI targeted-therapy (e.g. imatinib mesylate) has emerged as a new promising treatment approach for a subset of mastocytosis patients. *In vitro* studies on MC lines and human BM MCs have shown that imatinib inhibits phosphorylation of *KIT* and growth of MCs with wild-type *KIT* or mutations localized outside the activation loop (e.g. exon 17) of *KIT* such as the V560G, F522C, K509I or p.419del mutations [10–12, 27–29]; in contrast, the D816V *KIT* mutation confers constitutive resistance to the drug [30]. These preclinical findings correlated well with early reports showing response to imatinib in 18/26 patients (69%) who either lacked or were not screened for the D816V *KIT* mutation vs. 1/4 D816V-positive cases (25%) [12, 31–39]. These observations led to the approval by the FDA of imatinib for adult ASM patients who have no D816V *KIT* mutation or with unknown *KIT* mutational status; however, it should be noted that 10 out of those 18 responding cases previously evaluated by the FDA who were negative or not tested for the D816V *KIT* mutation, showed either the FIP1L1/PDGFRα fusion gene (*n* = 8) or juxtamembrane *KIT* mutations (*n* = 2), which might have contributed to an overestimation of the impact of the absence of the D816V *KIT* mutation itself in the response to imatinib in that series of patients.

To date, 121 mastocytosis patients, plus the 10 patients studied here, have been reported as being treated with imatinib for a total of 131 cases; from them, response to therapy data is publicly available for 128/131 patients (Table 2). The overall reported response rate to imatinib among patients with either no D816V *KIT* mutation or unknown *KIT* mutational status vs. D816V *KIT* mutation-positive patients is of 57% vs. 46%, respectively. However, highly heterogeneous response criteria have been used in different reports, e.g. while in some reports response to imatinib was defined by a substantially reduced degree of BM MC infiltration, in other series, patients were classified as responders whenever they showed improvement of symptoms related to the release of MC mediators after treatment. Such variability is due, at least in part, to the lack of consensus treatment response criteria for mastocytosis until 2007, when a first proposal was formulated [40]. More recently, specific response criteria for advanced mastocytosis have been redefined by the International Working Group–Myeloproliferative Neoplasms Research and Treatment (IWG-MRT) and the European Competence Network on Mastocytosis (ECNM) [41]; in turn, response criteria in terms of MC tumor load are still lacking for patients with non-aggressive (e.g. CM and ISM) forms of mastocytosis. In the trial here described, homogeneous and well-defined response criteria were used to evaluate both the reduction in BM MC burden and the improvement of symptoms related to the activation of MCs, as described above in detail. In addition, sequencing of exons other than exon 17 of *KIT* in highly-purified BM MCs [4, 23] allowed for reliable discrimination between those three cases who had *KIT* mutations involving exon 9 of *KIT* (e.g. K509I) and the seven patients with wild-type *KIT*.

**Table 2:** Response to imatinib in mastocytosis patients included in the present study and in previous reports

Year	Ref	Number of patients					Reported responses						Response referred to as MC cytoreduction					
		Total	WDSM features	Codon 816 KIT mutation	Exon 8-10 KIT mutations	PDGFR alterations	Type of response				Response rate		Type of response				Response rate	
							CR	PR	NR	NA	ORR‡	CRR	CR	PR	NR	NA	ORR‡	CRR
2003	[31]	1†	NA	NA	NA	NA	0	0	1	0	0/1	0/1	0	0	1	0	0/1	0/1
2003	[32, 33]	12†	NA	2	0	3	3	4	3	2	7/10	3/10	3	2	2	5	5/7	3/7
2004	[34]	1†	0	0	NA	1	1	0	0	0	1/1	1/1	1	0	0	0	1/1	1/1
2004	[35]	3†	NA	0	0	0	0	2	1	0	2/3	0/3	0	0	0	3	NA	NA
2004	[36]	1†	NA	1	NA	NA	0	0	1	0	0/1	0/1	0	0	1	0	0/1	0/1
2004	[10]	1†	1	0	1	NA	0	1	0	0	1/1	0/1	0	1	0	0	1/1	0/1
2004	[37]	9†	NA	2	NA	4	4	0	5	0	4/9	4/9	1	0	5	3	1/6	1/6
2005	[38]	1†	0	1	0	NA	1	0	0	0	1/1	1/1	1	0	0	0	1/1	1/1
2006	[12]	1†	1	0	1	NA	0	1	0	0	1/1	0/1	0	0	0	1	NA	NA
2006	[45]	14	NA	11	NA	1	1	10	3	0	11/14	1/14	0	3	6	5	3/9	0/9
2006	[46]	1	0	0	NA	1	1	0	0	0	1/1	1/1	0	0	0	1	NA	NA
2006	[47]	1	NA	0	NA	1	1	0	0	0	1/1	1/1	0	0	0	1	NA	NA
2007	[65]	1	NA	NA	NA	NA	0	0	1	0	0/1	0/1	0	0	1	0	0/1	0/1
2007	[63]	1	0	NA	NA	1	1	0	0	0	1/1	1/1	1	0	0	0	1/1	1/1
2007	[64]	1	NA	NA	NA	NA	0	1	0	0	1/1	0/1	0	1	0	0	1/1	0/1
2008	[62]	1	0	0	0	1	1	0	0	0	1/1	1/1	1	0	0	0	1/1	1/1
2008	[48]	1	0	0	NA	NA	0	1	0	0	1/1	0/1	0	0	0	1	NA	NA
2008	[49]	17	NA	NA	NA	NA	1	4	12	0	5/17	1/17	1	4	12	0	5/17	1/17
2008	[46]	1	NA	0	1	NA	0	1	0	0	1/1	0/1	0	0	0	1	NA	NA
2008	[39]	5†	NA	5	0	0	0	1	4	0	1/5	0/5	0	0	0	5	NA	NA
2008	[50]	5	0	3	NA	NA	0	2	3	0	2/5	0/5	0	0	0	5	NA	NA
2009	[66]	1	0	1	0	0	0	0	1	0	0/1	0/1	0	0	0	1	NA	NA
2009	[51]	20	0	13	NA	0	1	6	13	0	7/20	1/20	1	0	19	0	1/20	1/20
2011	[17]	1	1	0	1	NA	0	1	0	0	1/1	0/1	0	1	0	0	1/1	0/1
2011	[52]	1	0	0	NA	NA	0	1	0	0	1/1	0/1	0	0	0	1	NA	NA
2011	[53]	1	0	0	0	0	1	0	0	0	1/1	1/1	1	0	0	0	1/1	1/1
2012	[42]	2	0	0	1	NA	1	0	1	0	1/2	1/2	1	0	1	0	1/2	1/2
2012	[18]	1	1	0	0	NA	1	0	0	0	1/1	1/1	1	0	0	0	1/1	1/1
2012	[58]	1	0	0	0	0	0	0	1	0	0/1	0/1	0	0	1	0	0/1	0/1
2012	[67]	1	0	NA	NA	NA	0	0	1	0	0/1	0/1	0	0	1	0	0/1	0/1
2013	[16]	1	NA	0	1	0	0	0	0	1	NA	NA	0	0	0	1	NA	NA
2013	[54]	1	0	0	NA	NA	1	0	0	0	1/1	1/1	1	0	0	0	1/1	1/1
2013	[43]	2	NA	0	2	NA	2	0	0	0	2/2	2/2	0	0	0	2	NA	NA
2013	[55]	1	NA	0	NA	0	0	1	0	0	1/1	0/1	0	0	0	1	NA	NA
2013	[59]	1	0	0	0	0	0	0	1	0	0/1	0/1	0	0	1	0	0/1	0/1
2014	[14]	1	1	0	1	NA	1	0	0	0	1/1	1/1	1	0	0	0	1/1	1/1
2014	[15]	2	2	0	2	NA	2	0	0	0	2/2	2/2	2	0	0	0	2/2	2/2
2014	[60]	1	0	0	NA	0	0	0	1	0	0/1	0/1	0	0	1	0	0/1	0/1
2014	[56]	1	0	0	0	NA	0	1	0	0	1/1	0/1	0	0	0	1	NA	NA
2015	[57]	1	NA	0	0	0	0	1	0	0	1/1	0/1	0	0	0	1	NA	NA
2015	[61]	1	0	0	0	NA	0	0	1	0	0/1	0/1	0	0	1	0	0/1	0/1
2016	*	10	9	0	3	NA	4	1	5	0	5/10	4/10	4	1	5	0	5/10	4/10
	**Total**	**131**	**16**	**39**	**14**	**13**	**29**	**40**	**59**	**3**	**69/128**	**29/128**	**21**	**13**	**58**	**39**	**34/92**	**21/92**
											**(53%)**	**(23%)**					**(37%)**	**(23%)**

Ref, reference number in this manuscript; N, number of patients; WDSM, well-differentiated systemic mastocytosis; PDGFR, platelet-derived growth factor receptor; MC, mast cell; CR, complete response; PR, partial response; NR, no response; NA, not available; ORR, overall response rate; CRR, complete response rate.

*This study corresponds to the clinical trial here reported.

†Cases evaluated by the FDA. The study with reference number [39] was a manufacturer-sponsored clinical trial whose results were published after FDA approval.

‡ORR is defined as the proportion of patients who achieve CR or PR.

Overall, half of our cases showed response to imatinib, this consisting of CR by the recent ECNM consensus criteria [41] in all but one case. Interestingly, except for one patient with SM-CEL, CR was restricted to WDSM patients showing mutations in exon 9 (K509I) of *KIT*, whereas all non-responder cases had wild-type *KIT*. To date, 7 adult SM patients showing mutations at exons other than exon 17 of *KIT* (exons 9 and 10) have been reported to be treated with imatinib. Five patients had the K509I *KIT* mutation (3 ISM, 1 ASM and 1 MCL) [12, 14–16], 1 ASM patient had the F522C *KIT* mutation [10] and 1 MCL patient carried the p.A502-Y503dup at exon 9 [17]. One of the ISM patients carrying the K509I mutation had a concomitant GIST that did not respond to imatinib, and the response of ISM was not assessed/reported in this case [16]. Interestingly, SM and GIST also coexisted in our 3 familial cases who had the K509I mutation; in these 3 cases SM showed CR but the GISTs were unresponsive to imatinib. In the other 6 previously reported patients with exon 9 or 10 *KIT* mutations, CR or near CR of mastocytosis was reported [10, 12, 14, 15, 17]. Of note, all 6 responding patients reported previously, as well as the three K509I^+^ patients who achieved CR in our series, were diagnosed with WDSM or had features highly suggestive of such variant of SM (e.g. childhood onset, female gender, familial aggregation and mature-appearing, round shape CD25^−^ BM MCs), supporting the close association of mutations involving the extracellular membrane and transmembrane domains of the *KIT* gene with WDSM [19]. Similarly, objective responses to imatinib have been also reported in 3 children with CM and 1 adult patient with MC sarcoma showing deletion of codon 419 (p.419del) in exon 8 of *KIT*, a region located within the extracellular domain of the gene [42–44]. Altogether, these findings suggest that the presence of mutations involving the extracellular and transmembrane domains of *KIT* (exons 8 to 10) is strongly associated with response to imatinib in mastocytosis.

In contrast, our results also suggest that response to imatinib among SM patients who have wild-type *KIT* is limited, with only 1/6 cases achieving PR. Overall, 46 patients with mastocytosis lacking the D816V *KIT* mutation and other extracellular membrane/transmembrane *KIT* mutations who were treated with imatinib and further evaluated for response to therapy have been reported, with an overall response rate of 80% (37/46 cases) [18, 32–35, 37, 42, 45–62]. However, almost one third of such responding cases corresponded to patients with SM associated with either HES/CEL (*n* = 11/37) [32, 34, 37, 45–47] or chronic basophilic leukemia (*n* = 1/37) [68] carrying rearrangements of *PDGFR*α/*PDGFR*β; in turn, another 10/37 patients only showed improvement of MC-mediator release-associated symptoms and/or skin lesions [32, 48, 51, 52, 55, 57] Among the remaining 15 cases, 5 fulfilled criteria for CR [18, 49, 51, 53, 54] and 10 were reported to have PR [32, 45, 49, 50, 56]. It should be noted that unlike CR, which was defined in all reports by the disappearance of all mastocytosis-related signs and symptoms together with decrease of sT levels to < 20 μg/L, criteria used for establishing PR were more heterogeneous, a significant reduction of BM MC infiltrates being documented in only 4/10 PR patients [32, 45]. Altogether, these observations suggest that applying the more strict response criteria used in the present study, previously reported response of D816V-negative patients to imatinib therapy would probably had been lower. In fact, when only those patients who showed significant MC cytoreduction (e.g. ≥ 50%) after therapy are considered, and those with *PDGFR* rearrangements are excluded, the overall response rate to imatinib decreases to 25%, which is quite similar to that found in our clinical trial for this patient subgroup. A potential explanation for this low “true” response rate among patients who apparently carried wild-type *KIT* could be the use of inadequate methods (e.g. Sanger sequencing) for the detection of the D816V *KIT* mutation, particularly in cases with low MC burden.

In turn, only 5/34 (15%) adult mastocytosis patients who were not screened for the *KIT* mutation have been reported so far to respond to imatinib [31, 35, 63, 64]. Two of these patients only had transient improvement of symptoms [35], one had CM associated with HES and actually showed CR of HES with persistence of (cutaneous) mastocytosis lesions [31], one ASM patient with an associated eosinophilia and *PDGFR*β gene rearrangement achieved CR [63], and the remaining patient showed PR consisting of 50% reduction of BM MC counts after therapy [64]. Following the same considerations as described above for the D816V-negative patients, the “true” response rate (e.g. as defined by ≥ 50% MC cytoreduction) among patients with unknown *KIT* mutational status and no imatinib-sensitive mutations involving other genes (e.g. *PDGFR*) would decrease to only 3% (1/34 cases). Thereby, the estimated probability of lack of response to imatinib among patients who were not screened for *KIT* mutations is of 97%, which correlates with the expected frequency of exon 17 *KIT* mutations (∼94%) in SM, when highly-sensitive and robust PCR-based methods are used [4–7].

Although response to imatinib has been also reported in 13/28 patients (46%) with exon 17 *KIT* mutations [38, 39, 45, 50, 51], most of such responses were purely symptomatic, a significant reduction (e.g. ≥ 50%) in BM MC infiltration after therapy being documented in only 3 cases (11%) [38, 45]. Of note, among all such cases, the only patient who showed CR presented with an associated imatinib-sensitive BCR/ABL-positive CML [38].

In summary, our observations together with previous data from the literature suggest that the efficacy of imatinib in terms of reducing MC tumor load in SM patients lacking the D816V *KIT* mutation, relies on the existence of imatinib-sensitive genetic defects such as extracellular membrane/transmembrane *KIT* mutations or *PDGFR* gene rearrangements, rather than on the absence of the *KIT* D816V mutation by itself. Thus, the few responses reported in true D816V-negative patients who were only screened for mutations in exon 17 of the *KIT* gene, could be most likely related to the presence of already-known (unexplored) imatinib-sensitive *KIT* and/or *PDGFR* mutations, or still undiscovered imatinib-sensitive mutations in genes other than *KIT*. Importantly, since objective responses to imatinib have also been obtained in ISM [14, 15, 18, 45, 54] SSM [45], MCL [15, 17] and SM-AHNMD [32, 34, 37, 38, 51, 62, 63], it seems reasonable that imatinib therapy should not be restricted to patients with ASM, whenever imatinib-sensitive molecular alterations are detected.

## MATERIALS AND METHODS

### Inclusion and exclusion criteria

The inclusion criteria used in this phase IV, open-label, uncontrolled clinical trial with imatinib (clinicaltrials.gov #NCT01297777) were as follows: 1) age ≥ 18 years; 2) diagnosis of systemic MC disease according to the WHO 2008 diagnostic criteria [1] and more recent criteria for WDSM [19], and; 3) absence of exon 17 *KIT* mutation. According to such criteria, all adult patients fulfilling the WHO 2008 diagnostic criteria for SM (with or without WSDM) and those who met the WDSM criteria (even when the WHO 2008 diagnostic criteria were not strictly fulfilled in the presence of a significant MC burden in ≥ two tissues), were eligible for participating in the study, independently from the type and severity of MC mediator-release symptoms and the levels of sT, whenever mutations at exon 17 of the *KIT* gene were ruled out. For the purposes of this study, patients within the ASM, MCL or SM-AHNMD disease categories according to the WHO 2008 classification of mastocytosis are also referred to as having advanced disease, as proposed elsewhere [41]; otherwise, patients were considered as having non-advanced disease.

Additionally, the following exclusion criteria were used: 1) previous treatment with TKI; 2) serum positivity for the human immunodeficiency virus (HIV) or active viral hepatitis; 3) impaired liver function, defined as serum bilirubin levels ≥ 2 mg/dL and/or aspartate transaminase (AST) or alanine transaminase (ALT) ≥ 3 times upper normal limit; 4) impaired renal function, defined as serum creatinine ≥ 2 mg/dL; 5) grade ≥ 3 cytopenias not related to mastocytosis; 6) severe cardiopathy (grade III/IV of New York Heart Association –NYHA–, or left ventricular ejection fraction < 50%); 7) pregnancy or breastfeeding, and; 8) female patients not using contraceptive methods.

### Patients

Ten adult patients (2 males, 8 females) selected from a group of 453 consecutive SM patients followed at the Spanish Network on Mastocytosis (REMA) were enrolled in the study between January 2011 and August 2011. All patients were treated on an intention-to-cure basis, regardless of the prognostic impact of the specific subtype of mastocytosis or the severity of MC-mediator release-associated symptoms. Each patient gave his/her written informed consent to participate in the study, which was approved by the local institutional Ethics Committee (Complejo Hospitalario de Toledo, Toledo, Spain) where the trial took place.

### Screening for *KIT* mutations

Absence of the D816V *KIT* mutation and other exon 17 *KIT* mutations was confirmed in genomic DNA extracted from fluorescence-activated cell sorting (FACS)-purified BM MCs by a previously described peptide nucleic acid (PNA)-mediated polymerase chain reaction (PCR) technique [4]. Mutations involving other exons of *KIT* were also investigated by direct sequencing of PCR products using the Sanger method [23], such mutations being detected in 4/10 cases.

### Treatment protocol and follow-up schedule

Patients who showed > 30% BM MC infiltration by immunohistochemistry, advanced disease (defined by the presence of C-findings, > 20% of MCs in BM smears and/or an associated non-MC lineage hematological disease) and/or another malignant mastocytosis-associated disease (e.g. GIST), received 400 mg/day of imatinib orally for 12 months (*n* = 6/10); otherwise, a standard dose of 300 mg/day was administered for 12 months (*n* = 4/10). Among those patients selected to receive 400 mg/day of imatinib, treatment was discontinued whenever < 10% decrease in BM MC tumor load by flow cytometry had been achieved at month +6 of therapy. Potential treatment-related adverse events were closely monitored throughout the study and graded according to the National Cancer Institute Common Toxicity Criteria whenever they developed. In patients who had grade 3 or 4 adverse events, imatinib was either reduced by 100 mg or transiently stopped until the adverse event had resolved and reintroduced afterward at lower doses, respectively.

Blood tests including complete blood count and differential, routine biochemistry and sT were systematically performed before starting imatinib and at months +1, +2, +3, +6, +9 and +12 of therapy. Spleen and liver ultrasonography was performed in all cases before starting imatinib and, whenever organomegalies were detected, also at months +6 and +12 of therapy.

Both clinical evaluation and physical examination of patients were carried out by specialized medical doctors with expertise on mastocytosis (I.A-T, A.M and/or L.E) at months +3, +6, +9 and +12. Mastocytosis-related symptoms were evaluated in every patient by the same doctors using Likert-type scales obtained from specific questionnaires designed by the REMA, by which MC-mediator release-associated symptoms (e.g. pruritus, flushing, abdominal cramping, diarrhea and anaphylaxis) were individually graded according to their frequency (0 = absent; 1 = less than monthly; 2 = monthly; 3 = weekly; 4 = daily or almost daily) and degree of severity (1 = no therapy required; 2 = kept under control with antiH1; 3 = corticosteroids required; 4 = epinephrine and/or hospitalization required). Individual scores per symptom were calculated for the 3 months preceding the start of imatinib, and then at months +3, +6, +9 and +12 of therapy, by multiplying their frequency and severity values (Figure 3).

To evaluate the cutaneous response, skin biopsies were performed before starting imatinib and at months +6 and +12 of therapy in all patients who presented with mastocytosis in the skin. To evaluate changes in BM MC infiltration, a complete BM study including histology, cytology and flow cytometry, was performed before starting imatinib and at month +12 of therapy; an additional BM study was performed at month +6 of therapy in those patients who had > 30% BM MC infiltration and/or advanced disease at the moment of starting therapy. For both cytologic and histologic studies, all specimens were independently reviewed by three experts (I.A-T, L.E and M.M). For the evaluation of MCs by flow cytometry, all BM samples were analyzed in parallel in two different laboratories of the REMA by two and three independent experts, respectively (JM.M and L.S-M, and A.M, C.C and C.T, respectively).

### Endpoints and response criteria

The primary and secondary endpoints of the study were: 1) a change (e.g. decrease) in MC infiltration of involved organs including the BM and the skin, 2) a change (e.g. decrease) in the size of organomegalies (if present) as evaluated by abdominal ultrasonography, and 3) a change (e.g. decrease) in MC-mediator release-associated symptoms and sT levels. Complete response (CR) was defined as complete resolution of all disease signs and symptoms including BM MC infiltration, skin lesions, organomegalies and MC-mediator release-associated symptoms, plus a decrease in sT below normal values (< 11.5 μg/L). In turn, partial response (PR) was defined as ≥ 50% reduction in BM MC infiltration and improvement of skin lesions and/or organomegalies. All other patients were considered as non-responders (NR). Patients having outcomes other than CR, symptomatic response was evaluated as a separate endpoint using Likert-type scales obtained from specific questionnaires designed by the REMA.

All authors had access to clinical trial data and participated in data analysis, interpretation of results and writing, revision and final approval of the manuscript.

## Abbreviations

ALT: Alanine Transaminase
ASM: Aggressive systemic mastocytosis
AST: Aspartate Transaminase
BM: Bone marrow
CEL: Chronic eosinophilic leukemia
CM: Cutaneous mastocytosis
CML: Chronic myeloid leukemia
CR: Complete response
DNA: Deoxyribonucleic acid
ECNM: European Competence Network on Mastocytosis
FACS: Fluorescence-activated cell sorting
FDA: Food and Drug Administration
FGFR1: Fibroblast Growth Factor Receptor 1
G-CSF: Granulocyte colony-stimulating factor
GIST: Gastrointestinal stromal tumor
HES: Hypereosinophilic syndrome
HIV: Human Immunodeficiency Virus
HUMARA: Human androgen receptor assay
ISM: Indolent systemic mastocytosis
IWG-MRT: International Working Group on Myeloproliferative Neoplasms Research and Treatment
JAK: Janus kinase
MC: Mast cell
MCL: Mast cell leukemia
NR: No response
NYHA: New York Heart Association
PB: Peripheral blood
PCR: Polymerase chain reaction
PDGFR: Platelet-derived growth factor receptor
PNA: Peptide nucleic acid
PR: Partial response
REMA: Spanish Network on Mastocytosis
SM: Systemic mastocytosis
SM-AHNMD: Systemic mastocytosis with an associated clonal haematological non-mast cell disease
SSM: Smoldering systemic mastocytosis
sT: Serum tryptase
TK: Tyrosine kinase
TKI: Tyrosine kinase inhibitor
WDSM: Well-differentiated systemic mastocytosis
WHO: World Health Organization.
